# Single versus double blastocyst transfer in first and second frozen-thawed embryo transfer cycle in advance-aged women: a two-center retrospective cohort study

**DOI:** 10.1186/s12905-023-02753-x

**Published:** 2024-01-18

**Authors:** Yuxi Zhou, Hui Ji, Mianqiu Zhang, Juanjuan Zhang, Xin Li, Junqiang Zhang, Xiufeng Ling, Li Chen, Chun Zhao

**Affiliations:** 1 Department of Reproductive Medicine, Nanjing Women and Children’s Healthcare Hospital, Women’s Hospital of Nanjing Medical University, Nanjing, 210004 China; 2 Department of Reproductive Medicine, Changzhou Maternal and Child Health Care Hospital, Changzhou, 213000 China

**Keywords:** Single embryo transfer, Blastocyst transfer, Maternal age, Live birth

## Abstract

**Background:**

The present evidence is deficient for the trade-offs between the pros and cons of single blastocyst transfer (SBT) versus double blastocyst transfer (DBT) in frozen-thawed embryo transfer cycles for women in advanced reproductive age, especially in the second cycle. The current study aimed to investigate the impact of transferred blastocyst numbers on pregnancy outcomes in the first and second embryo transfer for women ≥ 35 years.

**Methods:**

This was a retrospective cohort study including 1284 frozen-thawed blastocyst transfer (FBT) cycles from two reproductive centers. We analyzed the pregnancy outcomes after SBT and DBT in the first and second FBT cycles. Moreover, stratified analysis was conducted by maternal age.

**Results:**

In the first FBT cycle, the LBR was higher in the DBT group than that in the SBT group [52.3% vs. 33.9%; adjusted odds ratio (aOR), 1.65; 95% confidence interval (CI), 1.26–2.15, *P* < 0.001]. However, the LBR of the DBT group showed no remarkable difference compared with that of the SBT group in the second cycle of FBT (44.3% vs. 33.3%; aOR, 1.30; 95% CI, 0.81–2.08; *P* = 0.271). Furthermore, stratified analysis by age showed a higher LBR for the DBT group than the SBT group in patients aged 38–42 years (43.1% vs. 33.9%; aOR, 2.27; 95% CI, 1.05–4.90; *P* = 0.036).

**Conclusions:**

The present study demonstrated that the SBT regimen is a better choice for both, the first and second frozen-thawed embryo transfer cycles, for women aged 35–37 years. Additionally, the DBT regimen is still recommended to achieve a high LBR in women aged 38–42 years in the second FBT cycle. These findings may be beneficial for deciding the embryo transfer regimens in women of advanced reproductive age.

## Background

Owing to the accumulation of clinical experience and advancement in cryopreservation techniques in the last two decades, the objective of assisted reproductive technology (ART) has switched from achieving better pregnancy rates to attaining comfortable and safe pregnancies [1]. Furthermore, there has been a growing number of frozen-thawed embryo transfer (FET) cycles. FET has been widely used in in-*vitro* fertilization (IVF) or intracytoplasmic sperm injection (ICSI) as it can effectively improve the cumulative pregnancy rate, avoid successive oocyte retrieval procedures, and allow embryos to be transferred to a more physiological uterine environment. Although the transfer of two or more embryos has a high chance of achieving pregnancy, it also increases the chance of multiple pregnancies, which can cause serious health risks for both mother and offspring [2, 3]. Reducing the number of transferred embryos, particularly through single blastocyst transfer (SBT), has been demonstrated to be the most effective method for decreasing the incidence of multiple pregnancies without compromising pregnancy outcomes [4, 5].

After 35 years of age, the overall euploid rate of blastocysts decreases significantly and it continues to decrease with increasing age [6]. The Practice Committee of the American Society for Reproductive Medicine (ASRM) suggests the first FET cycle as a favorable factor for determining the number of embryos transferred [7]. For older patients, to achieve pregnancy within a short period and to reduce the cost of multiple ART cycles, most clinicians and patients are unwilling to adopt the SBT technique. However, multiple embryo transfers increase pregnancy complications. The researchers are still trying to elucidate if there is a better embryo transfer regimen for women of advanced age. Although this is an intriguing topic, it demands further specific investigations.

Therefore, we retrospectively investigated the appropriate the FET strategy for women aged ≥ 35 years to provide more clarity to clinicians and patients on this issue.

## Methods

### Study population

A retrospective cohort study was carried out in two reproductive centers in China, namely, Women’s Hospital of Nanjing Medical University, and Changzhou Maternal and Child Health Care Hospital. We included all frozen-thawed blastocyst transfer (FBT) cycles in women aged ≥ 35 years (N = 1813) between January 2017 and January 2021. All procedures and protocols performed in this study were approved by the ethics committee of Women’s Hospital of Nanjing Medical University (NJFY-2020-KY-070) and the Ethics Committee of Changzhou Maternal and Child Health Care Hospital (2020 No.75) and were conducted based on the principles of the 1964 Helsinki Declaration and its later amendments or comparable ethical standards.

**Fig. 1:**
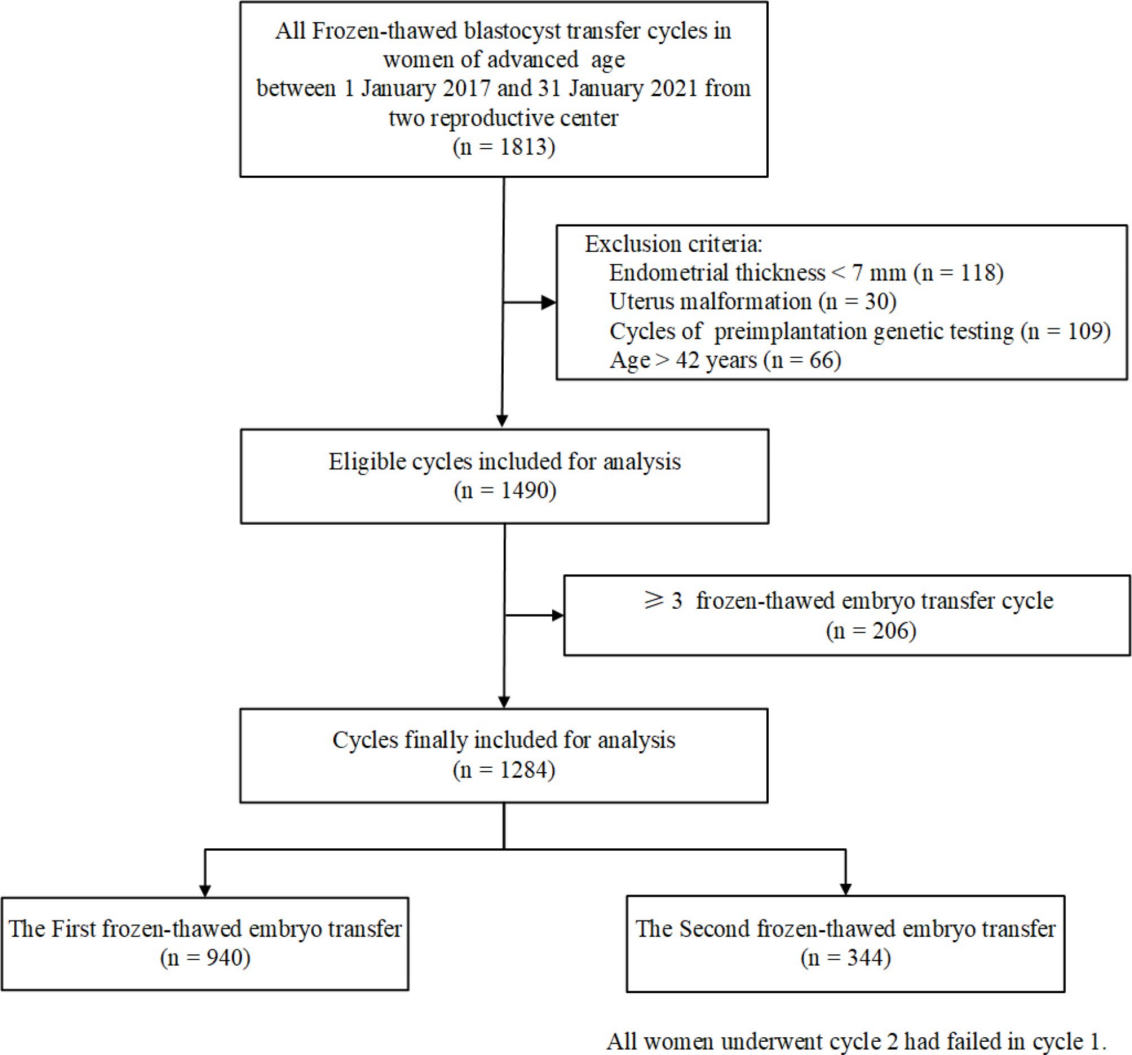
Flowchart of included and excluded cycles throughout the study

Eligible patients were 35–42 years old and underwent frozen-thawed autologous blastocyst transfer. The exclusion criteria were as follows: endometrium < 7 mm; evidence of a uterine malformation; cycles of preimplantation genetic testing (PGT); and ≥ 3 FET cycles. It is worth mentioning that all women included in this study that underwent cycle 2 had failed in cycle 1. Finally, a sum of 1,284 FBT cycles were included to conduct the investigation (Fig. 1).

### Ovarian stimulation

Patients received either a mild stimulation regimen or a flexible gonadotrophin-releasing hormone (GnRH) antagonist regimen. For old patients who underwent the mild stimulation regimen, 5 mg of letrozole (Letrozole tablets, Hengrui, China) or 50 mg of clomiphene (Fertilan, Medovhrmie Ltd., Republic of Cyprus) was administrated from day-3 to day-7 combined with 75 or 150 IU of recombinant follicle-stimulating hormone (rFSH) once a day. In the flexible GnRH antagonist regimen, gonadotropin (recombinant follicle-stimulating hormone, Gonal-F, Merck Serono, Switzerland) at 150–300 IU/day was started on the third day of menstruation and the GnRH antagonist (Cetrorelix 0.25 mg, Pierre Fabre Medicament Production, Aquitaine Pharm International, France) was added when a leading follicle was ≥ 14 mm in mean diameter. Human chorionic gonadotrophin at 10,000 IU (hCG, Lizhu Co., China) was injected to induce final oocyte maturation when at least 3 follicles were > 17 mm in mean diameter. After 35–36 h, the oocyte was retrieved. Oocytes were inseminated by regular in vitro fertilization (IVF) or intracytoplasmic sperm injection based on sperm quality. The embryos were cultured under our previously published protocol [8]. If there were at least three cleavage-stage embryos with good morphology (8 cells and < 20% fragmentation) on day 3, embryos were cultured to the blastocyst stage.

### Blastocyst grading

The blastocysts were graded based on Gardner’s scoring system [9], which involves blastocyst expansion, inner cell mass (ICM), and trophectoderm (TE). Blastocysts were evaluated on the expansion of the blastocyst cavity on a scale of 1–6. When the blastocyst arrived at a cavity expansion level 3 or over, ICM and TE were evaluated based on the size and density of the cells (A/B/C), respectively. A blastocyst evaluated 3 and above with an A or B for either ICM or TE was considered a good-quality embryo; if not, it was defined as a low-quality embryo (grades 3–6 AC/BC/CA/CB). Poor-quality blastocysts (grades 3–6 CC) were discarded due to their low developmental potential.

### Endometrial preparation for FET cycles and luteal phase support

The endometrial preparation regimen for FET cycles was selected by the physicians at their discretion. The modified natural cycle (NC) regimen was used for participants with regular menstrual cycles. In the modified NC regimen, ovulation was determined by using ultrasound to detect the leading follicle and measuring the progesterone levels. When the dominant follicle had reached at least 18 mm, endometrial thickness was 6 mm or thicker, and meanwhile, progesterone level was ≤ 1.5 ng/mL, 10,000 IU of hCG was administered as the ovulatory trigger. After ultrasound confirmation of ovulation, luteal phase support (LPS) was started by administering 10 mg thrice a day of progesterone (dydrogesterone, Abbott Biologicals B.V., The Netherlands). In artificial cycles, oral estrogen treatment was started from the second to the fourth day of the menstruation for one week, at a dose of 4–6 mg/day (estradiol valerate tablets, Progynova, Bayer, France), which was adjusted to 6–8 mg depending on the serum estradiol (E_2_) levels and endometrial thickness. After approximately ten days later, when endometrial thickness ≥ 7 mm and serum E_2_ ≥ 200 pg/mL, the patients were given 90 mg of vaginal progesterone (Crinone, Merck Serono, UK) once a day and 10 mg of dydrogesterone thrice a day (P + 1). Seven days after the administration of hCG or 5 days after the addition of progesterone, FET was performed. In all cases, LPS with progesterone was continued after embryo transfer. Once gestational sac and embryonic heartbeat were detected by transvaginal ultrasound, the LPS was maintained until 10–12 weeks of pregnancy.

### Clinical outcomes

The primary clinical outcome was the live birth rate (LBR) and the secondary.

clinical endpoints included clinical pregnancy rate (CPR), clinical miscarriage rate, and twin delivery rate (TDR). The delivery of a live infant after 28 weeks of pregnancy was considered a live birth. The presence of a gestational sac confirmed by transvaginal ultrasound 4 weeks after the transfer was considered a sign of clinical pregnancy. Miscarriage was defined as a loss of pregnancy before 20 weeks of gestation. TDR was calculated from the number of twin deliveries per FET cycle.

### Statistical analysis

The SPSS 26.0 software (IBM Corp., Armonk, NY, USA) was used to perform all statistical analyses. For continuous variables, the normality was assessed using QQ-plots and the Kolmogorov-Smirnov test. The data were analysed using student’s t-tests and presented as (for normally distributed data) or Mann-Whitney U tests (for non-normally distributed data). Continuous variables were expressed as mean ± standard deviation if they were normally distributed, while the median and interquartile range (IOR) were provided for non-normally distributed data. Pearson’s χ^2^ test or Fisher’s exact test was used to compare categorical variables, whose data are presented as proportions. A multivariate logistic regression analysis was used to assess the independent effect of the transferred blastocyst number on the LBR. The covariates included were: age, basal FSH, and the number of good-quality blastocysts transferred (1 or 2 vs. 0). The odds ratios (OR) or adjusted odds ratios (aOR) and 95% confidence intervals (CI) are used to display the results. Statistical significance was accepted at a P-value < 0.05. Hosmer-Lemeshow goodness-of-fit test was used to assess the goodness of fit of the model.

## Results

### Demographic and clinical characteristics of women

In total, 1284 FBT cycles were enrolled during the study period. The demographic and basal characteristics of women with live births and non-live births are presented in Table 1. Overall, the LBR per transfer was 44.7%. We observed that patients with a live birth were younger, had a lower basal serum FSH level (*P* = 0.003), higher proportion of first FET cycle, greater number of blastocysts transferred, and greater number of good-quality blastocysts transferred than those without a live birth (Table 1). Moreover, body mass index, cause of infertility, the type and duration of infertility, basal serum luteinizing hormone and E_2_ levels, endometrial thickness, and the type of endometrial preparation were not significantly different between those with and without a live birth. Women with SBT were more likely to have secondary infertility. There were no differences in the other baseline characteristics between those with SBT and DBT group (Table 2).

**Table 1:** Baseline characteristics of the patients with and without live birth

	Live birth (n = 574)	Non-live birth (n = 710)	*P*-value
Variables			
Age (years), n (%)			
35–37	410 (71.4)	403 (56.8)	< 0.001
38–42	164 (28.6)	307 (43.2)	
BMI (kg/m^2^), n (%)			0.309
< 18.5	25 (4.4)	44 (6.2)	
18.5–24.9	426 (74.2)	509 (71.7)	
≥ 25	123 (21.4)	157 (22.1)	
Cause of infertility, n (%)			0.173
Female infertility	343 (59.8)	384 (54.1)	
Male infertility	37 (6.4)	58 (8.2)	
Mixed infertility	121 (21.1)	176 (24.8)	
Unknown infertility	73 (12.7)	92 (13.0)	
Type of infertility, n (%)			0.472
Primary	159 (27.7)	184 (25.9)	
Secondary	415 (72.3)	526 (74.1)	
Duration of infertility (years)	6.00 (6.00–6.00)	6.00 (6.00–6.00)	0.217
Basal FSH (mUI/ml)	7.38 (6.27–8.52)	7.59 (6.44–8.95)	0.012
Basal LH (mUI/ml)	4.16 (3.19–5.52)	4.06 (3.00-5.50)	0.262
Basal E_2_ (pg/ml)	41.00 (28.00–57.00)	39.00 (28.00–54.00)	0.322
FET cycles rank			0.017
1st cycle	439 (76.5)	501 (70.6)	
2nd cycle	135 (23.5)	209 (29.4)	
N of blastocyst transferred, n (%)			< 0.001
1	223 (38.9)	362 (51.0)	
2	351 (61.1)	348 (49.0)	
N of good-quality blastocyst, n (%)			< 0.001
0	112 (19.5)	282 (39.7)	
1 or 2	462 (80.5)	428 (60.3)	
Endometrial thickness (mm)	9.00 (8.00-10.50)	9.00 (8.00–10.00)	0.048
Endometrium preparation, n (%)			0.829
NC	89 (15.5)	107 (15.1)	
AC	485 (84.5)	603 (84.9)	

*N* Number, *BMI* Body mass index, *NC* Natural cycle, *AC* Artificial cycles

**Table 2:** Comparison of Demographics between those patients with single blastocyst transfer and double blastocyst transfer

	SBT (n = 585)	DBT (n = 699)	*P*-value
Variables			
Age (years), n (%)			
35–37	355 (60.7)	458 (65.5)	0.073
38–42	230 (39.3)	241 (34.5)	
BMI (kg/m^2^), n (%)			0.702
< 18.5	34 (5.8)	35 (5.0)	
18.5–24.9	420 (71.8)	515 (73.7)	
≥ 25	131 (22.4)	149 (21.3)	
Cause of infertility, n (%)			0.240
Female infertility	334 (57.1)	393 (56.2)	
Male infertility	50 (8.5)	45 (6.4)	
Mixed infertility	123 (21.0)	174 (24.9)	
Unknown infertility	78 (13.3)	87 (12.4)	
Type of infertility, n (%)			< 0.001
Primary	116 (19.8)	227 32.5)	
Secondary	469 (80.2)	472 (67.5)	
Duration of infertility (years)	6.00 (6.00–6.00)	6.00 (6.00–6.00)	0.082
Basal FSH (mUI/ml)	7.58 (6.38–8.88)	7.43 (6.36–8.67)	0.197
Basal LH (mUI/ml)	4.00 (3.06–5.39)	4.23 (3.13–5.61)	0.084
Basal E_2_ (pg/ml)	44.00 (28.19-54.00)	40.00 (28.00–57.00)	0.405
Cycle rank			0.774
1	426 (72.8)	514 (73.5)	
2	159 (27.2)	185 (26.5)	
N of good-quality blastocyst, n (%)			0.078
0	194 (32.2)	200 (28.6)	
1 or 2	391 (66.8)	499 (71.4)	
Endometrial thickness (mm)	9.00 (8.00–10.00)	9.00 (8.00-10.50)	0.522
Endometrium preparation, n (%)			0.564
NC	93 (15.9)	103 (14.7)	
AC	492 (84.1)	596 (85.3)	

*SBT* single blastocyst transfer, *DBT* double blastocyst transfer, *N* Number, *BMI* Body mass index, *NC* Natural cycle, *AC* Artificial cycles

### Clinical outcomes

The clinical outcomes of the first and second FET cycles are presented in Table 3. In cycle 1, the LBR (52.3% vs. 39.9%; OR, 1.65; 95% CI, 1.28–2.14) and CPR (64.2% vs. 50.2%; OR, 1.78; 95% CI, 1.37–2.31) were significantly higher in the double blastocyst transfer (DBT) group than in the SBT group. The differences were statistically significant after multiple regression analysis (LBR: aOR, 1.65; 95% CI, 1.26–2.15; CPR: aOR, 1.78; 95% CI, 1.36–2.32). The confounding factors considered in the regression model were maternal age, basal FSH level, and quantity of good-quality blastocysts transferred. There were no statistical differences between the two groups in terms of the miscarriage rate. In cycle 2, LBR did not differ significantly between the two groups (44.3% vs. 33.3%; aOR, 1.30; 95% CI, 0.81–2.08) after adjusting for confounding factors (Table 3).

**Table 3:** Pregnancy outcomes of the First and second FET cycle

Cycle rank							
Cycle 1		SBT (n = 426)	DBT (n = 514)	Crude OR (95% CI)	*P*-value	Adjusted OR (95% CI) ^*^	*P*-value
	Live birth rate	170/426 (39.9)	269/514 (52.3)	1.65 (1.28–2.14)	< 0.001	1.65 (1.26–2.15^) a^	< 0.001
	Clinical pregnancy rate	214/426 (50.2)	330/514 (64.2)	1.78 (1.37–2.31)0	< 0.001	1.78 (1.36–2.32)	< 0.001
	Miscarriage rate	45/214 (21.0)	66/330 (20.0)	0.94 (0.61–1.44)	0.771		
Cycle 2		SBT (n = 159)	DBT (n = 185)	Crude OR (95% CI)	*P*-value	Adjusted OR (95% CI) ^*^	*P*-value
	Live birth rate	53/159 (33.3)	82/185 (44.3)	1.52 (1.03–2.26)	0.037	1.30 (0.81–2.08) ^b^	0.271
	Clinical pregnancy rate	65/159 (40.9)	107/185 (57.8)	1.98 (1.29–3.05)	0.002	1.63 (1.03–2.60)	0.039
	Miscarriage rate	12/65 (18.5)	26/107 (24.3)	1.42 (0.66–3.05)	0.371		

*SBT* Single blastocyst transfer, *DBT* Double blastocyst transfer, *OR* Odds ratio, *CI* Confidence interval

^*^Adjusted for age, basal FSH, and N of good-quality blastocyst transferred (1 or 2 vs. 0)

^a^P value = 0.883 for Hosmer-Lemeshow goodness-of-fit test, which did not indicate significant poor fit

^b^P value = 0.370 for Hosmer-Lemeshow goodness-of-fit test, which did not indicate significant poor fit

### Analysis stratified by maternal age

We analyzed the association between transferred blastocyst number (2 vs. 1) and LBR in two subgroups stratified by age, i.e., 35–37 years and 38–42 years (Table 4). For patients aged 35–37 years, the LBR of the DBT group was considerably higher than that of the SBT group in the first cycle (aOR, 1.75; 95% CI, 1.27–2.43). However, the LBR of DBT did not increase in the second cycle (aOR, 0.94; 95% CI, 0.52–1.70). For patients aged 38–42 years, the LBR did not improve significantly in the DBT group compared with the SBT group in the first cycle (aOR, 1.45; 95% CI, 0.91–2.31). Meanwhile, we found the DBT group had significantly higher LBR than the SBT group in the second cycle (aOR, 2.27; 95% CI, 1.05–4.90). The confounding factors considered in the regression model were basal FSH level and the number of good-quality blastocysts transferred.

**Table 4:** Logistic regression analysis for live birth in DBT cycles compared with SBT cycles stratified by maternal age

Age (years)	Cycle rank	Group	N miscarriages/N cycles (MR per cycle, %)	N live-births/N cycles (LBR per cycle, %)	Adjusted OR (95% CI) ^*^	*P*-value
35–37	Cycle 1 (n = 616)	SBT	30/271 (11.1)	121/271 (44.6)	Reference	0.001
		DBT	44/345 (12.8)	201/345 (58.3)	1.75 (1.27–2.43) ^a^	
	Cycle 2 (n = 197)	SBT	7/84 (8.3)	37/84 (44.0)	Reference	0.936
		DBT	15/113 (13.3)	51/113 (45.1)	0.94 (0.52–1.70) ^b^	
38–42	Cycle 1 (n = 324)	SBT	15/155 (9.7)	49/155 (31.6)	Reference	0.122
		DBT	22/169 (13.0)	68/169 (40.2)	1.45 (0.91–2.31) ^c^	
	Cycle 2 (n = 147)	SBT	5/75 (6.7)	16/75 (21.3)	Reference	0.036
		DBT	11/72 (15.3)	31/72 (43.1)	2.27 (1.05–4.90) ^d^	

*N* Number, *LBR* live birth rates, *MR* miscarriage rate, *SBT* Single blastocyst transfer, *DBT* Double blastocyst transfer, *OR* Odds ratio, *CI* Confidence interval

^*^Adjusted for basal FSH and N of good-quality blastocyst transferred (1 or 2 vs. 0)

^a^P value = 0.825 for Hosmer-Lemeshow goodness-of-fit test, which did not indicate significant poor fit

^b^P value = 0.929 for Hosmer-Lemeshow goodness-of-fit test, which did not indicate significant poor fit

^c^P value = 0.612 for Hosmer-Lemeshow goodness-of-fit test, which did not indicate significant poor fit

^d^P value = 0.372 for Hosmer-Lemeshow goodness-of-fit test, which did not indicate significant poor fit

**Fig. 2:**
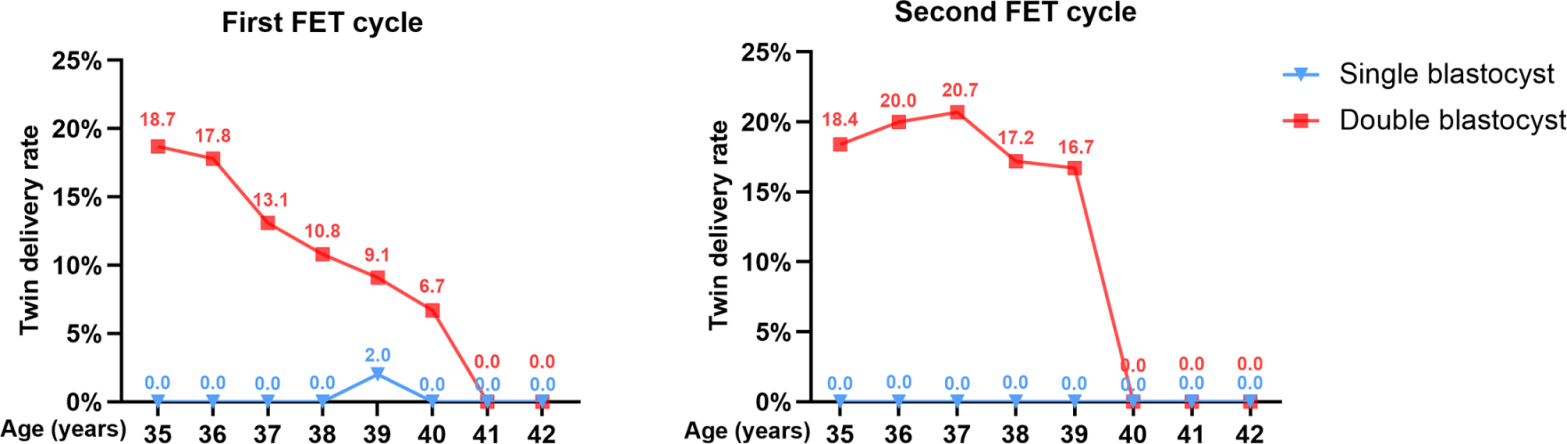
Twin delivery rates for each age. **A** the first frozen embryo transfer cycle; **B** the second frozen embryo transfer cycle

We then calculated the TDR for each age. We found that the TDR was higher in the DBT group (9.1–20.7%) compared with the SBT group (0.0–2.0%) for women aged 35–39 years (Fig. 2). The TDR was almost 0% for patients aged 40–42 years in both, cycle 1 and cycle 2.

## Discussion

To the best of the author’s knowledge, our study is the first study to focus on the association between the rank of FET cycles and the number of blastocysts transferred in women of advanced reproductive age. Our findings revealed that DBT was associated with higher odds of live births than SBT in women aged 35–37 years in the first FET cycle and women aged 38–42 years with previously failed FET cycles. The TDR was almost zero in patients aged 40–42 years, irrespective of the cycle rank and the number of blastocysts transferred.

Previous studies have reported that single embryo transfer (SET) is not suitable for all women. Therefore, the SET regimen is not recommended for women older > 40 years [10]. Fujimoto et al. reported that the CPR and LBR were significantly lower after the SET regimen than the double embryo transfer (DET) regimen in women aged 37–40 years [11]. The latest guidelines by ASRM on the limit of the number of embryos transferred recommend less than three cleavage-stage embryos or blastocysts with unknown euploidy for women > 35 years [7], without specifying the exact number of embryos. However, multiple pregnancies could increase maternal and perinatal morbidity and mortality and further increase medical costs [12–14]. Moreover, there is a lack of evidence on the pros and cons of SBT and DBT regimens for older patients, and clinicians wonder whether the DBT regimen should be avoided to reduce the risk of multiple pregnancies regardless of patients’ desire to achieve pregnancy. The SBT regimen is longer than the DBT regimen for similar cumulative pregnancy outcomes [11], which is a remarkable disadvantage for women of advanced age. Currently, there is no consensus on how older women should choose the number of blastocysts to be transferred. Therefore, our study evaluated whether women between the age of 35 and 42 should receive single or double blastocysts.

We assumed that the first choice of all women of advanced age in cycle 1 is SBT. The TDR was significantly high in the DBT group in patients aged 35–37 years, and the LBR with the DBT regimen was comparable to SBT in women aged ≥ 38 years. Furthermore, when the first FET cycle fails, DBT can be considered for women aged 38–42 since the LBR was notably higher in the DBT group than in the SBT group. Additionally, although the TDR was still high after DBT in women aged 38–39, this group could obtain live births faster or restart their next oocyte retrieval cycle. Advanced maternal age is relevant to a decline in fertility and lower FET success rates, but one problem that cannot be ignored is cost-effectiveness (in terms of time and money). The extra time needed for multiple frozen-thawed SET may lead to the additional loss of fertility potential [15]. An earlier study reported that one fresh SET, followed by an extra frozen-thawed SET, is cost-effective in women less than 32 years of age compared with the DET regimen. In patients > 33 years, the DET regimen is more effective but it is costlier than the SET regimen [16].

Some studies have explored the effect of transferred blastocyst numbers on pregnancy outcomes in women stratified by age. An earlier study reported a similar pregnancy rate and a lower multiple pregnancy rate in the elective SBT (eSBT) regimen compared with the DBT regimen in women aged 35–39 years [17]. Some studies analyzed the clinical outcomes following eSBT and DBT regimens and the findings revealed a similar LBR in both cohorts [18, 19], but the eSBT regimen had a lower risk of multiple pregnancies in populations of all ages for both, fresh and vitrified-thawed cycles. However, the blastocyst quality was not analyzed in these studies. In 2020, Chen et al. [4] evaluated the pregnancy outcomes related to different amounts and qualities of transferred blastocysts in age-stratified women (≥ 35 years and < 35 years), and concluded that the eSBT regimen is an ideal alternative for patients of any age when good-quality blastocysts exist. Nonetheless, the aforementioned studies on advanced maternal age did not discuss the effect of FET cycle rank (first or second) on the choice of embryo transfer regimen. Our results were partly consistent with previous studies. In the first cycle of FET, DBT regimen has a higher LBR, but a higher TDR makes SBT a better choice. When the first cycle of FET fails, for women aged 35–37, DBT has a similar LBR as SBT. However, for women aged 38–42 years, a different embryo transfer strategy was needed for the first and second FET cycles. When the first cycle of FET fails, the DBT regimen should be decisively selected to achieve higher LBR.

A randomized trial reported similar LBR between eSBT and DBT regimens if PGT was used [20]. A previous study has reported euploid blastocyst rates in females aged 35–37 years, 38–40 years, and 41–42 years as 49.7%, 42.8%, and 28.2%, respectively [6]. Moreover, embryo aneuploidy and abortion rates are known to increase with maternal age [21]. Our study excluded the PGT cycle; however, the question arises, when the euploidy of the blastocyst is unknown, can the DBT regimen be more reasonable for older patients? Indeed, the DBT regimen led to significantly improved LBR for women aged 38–42 in cycle 2 in stratified analysis. Moreover, the TDR of women aged ≥ 40 years is low. Clinicians in some countries have voluntarily embraced the SBT regimen as the standard practice for IVF since it is often covered in the national healthcare insurance for IVF treatments [22, 23]. However, ART and PGT treatments are not covered by medical insurance in China. Since all costs are borne by patients, they financially burden them and thereby lead to the reduced willingness of selecting PGT as an option to achieve pregnancy.

The study has several advantages. A major strength is the population-based design and relatively large sample size of elderly infertile women. In addition, this is the first study to assess the relationship between cycle rank and the number of blastocysts transferred in advanced maternal age. Inevitably, there are some limitations to this study. (1) The retrospective nature of the study suggests that selection bias could not be avoided. (2) There was insufficient sample size to draw sufficiently compelling conclusions. (3) Owing to the limited number of cycles, our findings require verification in additional studies that involve more older women, including those who have had previously unsuccessful FET cycles and those who have a shortage of good-quality blastocysts.

## Conclusions

In conclusion, our findings indicate that the number of blastocysts transferred to women aged 35–42 years should be determined by age as well as the FET cycle rank. We believe that our data will help clinicians and patients arrive at the best decisions to achieve pregnancy. Furthermore, when the blastocyst euploidy is unknown, only one blastocyst should be transferred in women aged 35–37 years, and the DBT regimen should be followed for women > 37 years in the second FET cycle.

## Data Availability

The data that support the findings of this study are available from the corresponding authors upon reasonable request.

## Abbreviations

SBT: Single blastocyst transfer
DBT: Double blastocyst transfer
FBT: Frozen-thawed blastocyst transfer
ART: Assisted reproductive technology
ASRM: American Society for Reproductive Medicine
FET: Frozen-thawed embryo transfer
FBT: Frozen-thawed blastocyst transfer
PGT: Preimplantation genetic testing
GnRH: Gonadotrophin-releasing hormone
hCG: Human chorionic gonadotrophin
IVF: In *vitro* fertilization
ICM: Inner cell mass
TE: Trophectoderm
FSH: Follicle-stimulating hormone
LH: Luteinizing hormone
E_2_: Estradiol
OR: Odds ratio
CI: Confidence intervals
NC: Natural cycle
LPS: Luteal phase support
LBR: Live birth rate
CPR: Clinical pregnancy rate
TDR: Twin delivery rate
SET: Single embryo transfer
DET: Double embryo transfer
eSBT: Elective single blastocyst transfer
